# Seconds Matter: Rapid Non-Contact Monitoring of Heart and Respiratory Rate from Face Videos

**DOI:** 10.3390/s26051506

**Published:** 2026-02-27

**Authors:** Taha Khan, Péter Pál Boda, Annette Björklund, Stefan Malmberg

**Affiliations:** 1 Detectivio AB, 41390 Gothenburg, Sweden; peter@detectivio.com (P.P.B.); annette.bjorklund@detectivio.com (A.B.); 2 School of Public Health and Community Medicine, Institute of Medicine, Sahlgrenska Academy, University of Gothenburg, 40530 Gothenburg, Sweden; stefan@detectivio.com

**Keywords:** non-contact vital signs, remote photoplethysmography, heart rate, respiratory rate, Eulerian video magnification

## Abstract

Accurate, non-contact vital-sign monitoring promises a scalable alternative to conventional sensors, yet low signal quality and long recording times have limited real-life adoption. We present a dual-modality system that combines Eulerian video magnified remote photoplethysmography (rPPG) from facial videos with optical flow-based shoulder tracking to estimate heart rate (HR) and respiratory rate (RR) from ultra-short 15 s recordings. With 200 participants, each providing 2 videos, 387 videos passed strict usability criteria, excluding flicker, blur, occlusion, and low illumination. For 15 s recordings, the HR estimates reached 98.5% accuracy within a ±10 beats per minute tolerance (MAE = 3.25, RMSE = 4.88, r = 0.93; *p* < 0.05) and the RR estimates achieved 98.4% accuracy within a ±5 respirations per minute tolerance (MAE = 0.69, RMSE = 0.87, r = 0.90; *p* < 0.05), exceeding prior studies that required 30 to 60 s recording lengths. Computational analysis on a standard home computer confirmed feasibility, with near real-time performance achievable on optimized hardware. By integrating complementary modalities and rigorous video quality control, the system overcomes low-SNR challenges, delivering high-fidelity, clinically validated vital signs monitoring. These results establish a robust, scalable, and precise framework for clinical and home care, demonstrating that accurate, contact-free HR and RR monitoring can now be achieved in seconds, making rapid, real-life vital signs assessment practical and accessible.

## 1. Introduction

The vital signs (heart rate (HR), respiratory rate (RR), blood pressure, oxygen saturation (SpO_2_), and body temperature) are fundamental indicators of human physiology and are widely used to assess disease severity, trajectory, and mortality risk across a broad range of acute and chronic conditions. Measuring vital signs is the first and most decisive step in emergency care. Rapid HR and RR assessment drives timely clinical action, while repeated checks provide an early warning system for patient deterioration. In acute scenarios, such as hemodynamic instability or respiratory compromise, prompt recognition and intervention can be lifesaving. From the moment a patient arrives in the emergency department, every second counts. Despite its importance, vital signs assessment can be time-intensive, particularly under high workload or limited staffing. A multi-site time and motion study in four acute hospitals in England found that a complete vital signs assessment, including equipment setup, measurement, documentation, and recovery, averaged 5 min and 1 s (s) per patient. When interruptions were considered, this increased to 6 min and 26 s. Estimates were consistent across different institutions and staffing contexts [1]. These baseline durations are further magnified under pandemic conditions. Since COVID-19, enhanced infection control protocols require thorough disinfection of shared monitoring equipment, adding minutes per assessment. Observational data indicate that cleaning shared equipment significantly delays workflow; for example, disinfecting a medication trolley took on average 3 min and 53 s in a hospital simulation study. When repeated across many patient encounters, these demands sharply reduce throughput in high-acuity settings [2].

Beyond the substantial time burden associated with vital signs assessment, conventional contact-based monitoring carries additional limitations, including skin irritation, patient discomfort, restricted mobility, poor compliance, and risk of cross-contamination. These drawbacks have fueled research into non-contact and remote physiological monitoring technology that eliminates the need for wires and surface sensors, enabling continuous, hygienic, and scalable vital signs acquisition [3]. Among available modalities such as radar or thermal imaging, video-based monitoring using standard RGB cameras has emerged as the most accessible and clinically viable. This approach leverages remote photoplethysmography (rPPG), which detects subtle, periodic variations in skin reflectance to infer cardiovascular and respiratory activity.

rPPG is an optical technique that enables non-contact monitoring of blood-volume changes by analyzing subtle variations in light reflected from the skin under ambient or controlled illumination [4]. These variations are synchronized with the cardiac cycle, i.e., during systole, arterial blood volume increases, leading to greater light absorption and reduced reflectance, whereas during diastole, decreased blood volume produces the opposite effect, resulting in faint, rhythmic fluctuations in skin color [5]. High-speed cameras capture these variations across RGB channels, and advanced signal-processing algorithms reconstruct the pulse waveform to compute HR. A central challenge of rPPG lies in the extremely low amplitude of the pulsatile signal relative to the baseline reflectance, making it highly susceptible to noise. Key sources of interference include changes in ambient illumination, for example, flickering lights or shifts in lighting conditions, subject motion such as head movement, facial expressions, blinking, and inter-individual differences in skin tone and perfusion, all of which reduce the signal-to-noise ratio (SNR) [6,7,8]. Consequently, robust algorithms must accurately isolate the weak pulsatile component from the dominant background noise to provide reliable HR measurements. By overcoming the limitations of contact-based monitoring, non-contact, video-based rPPG systems can offer a practical, hygienic approach for rapid and continuous vital signs assessment, particularly valuable in emergency and high-acuity clinical settings.

RR estimation from video is inherently more challenging than HR because the respiratory component of the rPPG signal is weak and easily masked by noise. Pulse-derived respiratory methods extract RR by analyzing baseline wander and amplitude modulation in the rPPG signal, but they rely on a clean waveform and suffer greatly when exposed to motion or illumination artifacts [9]. In contrast, motion-based respiratory techniques track physical displacement of the chest or upper torso using optical flow or motion-magnification algorithms, directly capturing breathing-induced motion in a selected region of interest (ROI) [10]. By focusing on anatomical areas that move primarily with respiration, motion-based methods provide more robust and reliable RR estimates than pulse-derived methods under realistic, dynamic conditions, though careful ROI selection is still required to minimize interference from non-respiratory motion.

Advances in video-based HR estimation have employed Eulerian video magnification (EVM) to amplify subtle temporal variations in facial videos that correspond to cardiac activity [11]. EVM decomposes the video into spatial frequency bands and selectively amplifies temporal changes in the HR frequency range, thereby enhancing color fluctuations that reflect underlying blood flow. In some implementations, these amplified color variations can be analyzed in different color spaces, including intensity or chromatic components, to reconstruct a pulse waveform [12]. However, while EVM is powerful in controlled settings, it remains computationally expensive and highly sensitive to motion [12,13].

To improve robustness and reduce dependence on manual signal selection, subsequent rPPG methods applied blind-source separation techniques such as independent component analysis and principal component analysis. Independent component analysis treats the RGB time-series as a linear mixture of independent sources, including pulse, motion, illumination, etc., and decomposes them to recover the component exhibiting cardiac periodicity [14]. However, this method can suffer from ambiguities in component ordering and scaling, particularly under motion or illumination changes, which degrade signal quality. Principal component analysis, by contrast, projects the RGB signals into orthogonal components ranked by variance, if noise dominates the high-variance components while the pulsatile signal resides in a lower-variance direction [15]. Albeit this method is also sensitive to non-rigid motion, as facial movements can contaminate components, blend physiological and noise signals, and reduce the accuracy of HR estimation.

Model-based, color-invariant approaches were introduced to address these limitations by explicitly projecting the RGB signal into a space that separates the cardiac signal from luminance variations. The CHROM method projects the RGB signal onto a chrominance plane orthogonal to skin tone, effectively reducing the impact of global brightness changes [16]. The plane-orthogonal-to-skin (POS) approach refines this idea by computing a projection vector specifically aligned orthogonal to the skin-color manifold, improving robustness to varying illumination [17]. These model-based methods enhance performance in realistic lighting conditions, but challenges persist, particularly for individuals with darker skin tones (Fitzpatrick scale > 4) [18], where lower reflectance reduces the SNR, and for dynamic conditions, where non-linear motion artifacts remain difficult to compensate for.

In parallel, deep learning approaches have emerged as powerful tools for video-based HR estimation. End-to-end networks, such as RhythmNet and PhysNet, learn spatial and temporal features directly from raw video frames, bypassing multi-step preprocessing pipelines [19,20]. These models achieve high accuracy in controlled datasets with mean absolute error (MAE) often below 1 beat per minute (bpm), but their performance can degrade significantly in real-world clinical environments due to domain shifts, patient motion, and variations in camera properties. Furthermore, deep networks function as black boxes and are computationally demanding, raising practical concerns for real-time deployment in emergencies or high-acuity settings.

Recent evidence supports the feasibility of contactless rPPG monitoring in real-world and clinical settings, though validation is still to be provided. A large hospital-based study involving 1045 patients undergoing pulmonary testing demonstrated strong intraclass correlation agreement of 0.88 between camera-based rPPG HR and electrocardiogram over 60 s windows, supporting its clinical reliability [21]. In pediatric care, a prospective two-phase study with children aged ≤16 years found good Spearman correlation for HR (r = 0.82) in adolescents aged 12–16, although younger children showed greater discrepancies [22]. In older adults, a study of elderly subjects using consumer RGB cameras reported feasible HR estimation via rPPG under realistic conditions [23]. Non-contact vital sign monitoring has been clinically validated with 30 surgical patients, where a webcam-based rPPG system measured heartbeat intervals that strongly agreed with a conventional patient monitor (r = 0.8) across different stages of anesthesia [24]. Finally, in a preoperative clinical population (*n* = 200), a prospective feasibility study recently explored rPPG-based blood pressure and hemoglobin estimation, demonstrating how rPPG technology can be adapted to complex hospital workflows and diverse patient demographics [25]. Overall, these studies highlight that while rPPG technology has matured significantly, further research is needed to optimize latency, motion robustness, and clinical integration before widespread adoption in high-acuity settings.

In the case of RR, the clinical validation of RR estimation with RGB cameras shows promising accuracy metrics. In a large hospital-based trial with 963 patients, a rPPG system measured RR over 60 s, and Bland–Altman analysis indicated that 96.0% of measurements fell within the 95% limits of agreement (LoA) compared to clinical reference, with a mean difference of approximately 0.7 respirations per minute (rpm) [26]. In a separate perioperative study of 216 patients using video plethysmography, the estimated RR correlated strongly with manual counting (r = 0.87), and the bias was 1.40 ± 1.96 rpm, with a success rate of 99.1% for the method [27]. These results suggest that RGB-camera methods can estimate RR with clinically acceptable accuracy, although motion robustness and real-time performance remain important challenges for broad deployment in high-acuity settings.

Despite progress in video-based vital signs estimation, significant challenges persist. Pulse-derived HR and RR methods remain highly sensitive to noise and motion, while motion-based RR approaches, though more robust, can still be affected by non-respiratory movements. Clinical evaluations reveal ongoing limitations compounded by limited demographic representation and reliance on multi-sensor setups. These issues highlight the need for a unified, accurate, and computationally efficient system capable of reliable vital signs monitoring in dynamic clinical environments.

To address these limitations and build on our earlier proof-of-concept study with patients suspected of COVID-19 [28], this work presents a unified and clinically validated framework for accurate, non-contact estimation of HR and RR using a standard RGB camera. HR is extracted from facial skin using the Q-channel rPPG signal, which consistently provides the highest SNR among chrominance components. This high SNR allows the HR pipeline to operate with minimal filtering, enabling fast execution on edge hardware while the Q channel’s inherent suppression of illumination fluctuations ensures reliable HR estimation. RR is computed from a complementary motion-based analysis of shoulder displacement, a strategy that isolates respiratory motion while minimizing the influence of facial movement and lighting changes. Across a large clinical cohort, the combined system achieved nearly 99% accuracy for both HR and RR and maintained processing speeds suitable for near real-time operation on resource-limited devices. Overall, this integrated framework resolves key challenges related to robustness, generalizability, and computational efficiency, advancing camera-based vital signs monitoring toward practical and scalable clinical use.

## 2. Materials and Methods

This section details the hardware configuration, data acquisition protocol, and the dual signal processing pipelines for contactless HR and RR estimation, followed by the statistical methods used to assess their accuracy and agreement with reference measurements.

### 2.1. Study Design, Setting, and Ethics

The present study is part of the ongoing “Remote Investigation and Assessment of Vital Signs” (RIA-VS) clinical investigation (universal trial number: U1111-1261-8273, and ClinicalTrials.gov ID: NCT05022264) [28]. The study is approved by the Swedish Medical Products Agency and the Swedish Ethical Review Authority, conducted in accordance with the Declaration of Helsinki, the principles of Good Clinical Practice (ICH-GCP), and applicable Swedish regulations for clinical investigations of medical devices. The study follows a method-comparison design to evaluate the agreement between contactless vital sign measurements obtained with the investigational system and conventional reference methods for HR and RR. Adult patients (≥18 years) are recruited in routine primary care, where they undergo standard-of-care vital sign assessment and brief video recording by an independent clinical research team. All participants provide written informed consent before any study-specific procedures. Participation does not influence clinical management. All diagnostic and therapeutic decisions are based solely on vital signs obtained using standard contact-based equipment.

### 2.2. Systems Architecture and Experimental Setup

**Figure 1:**
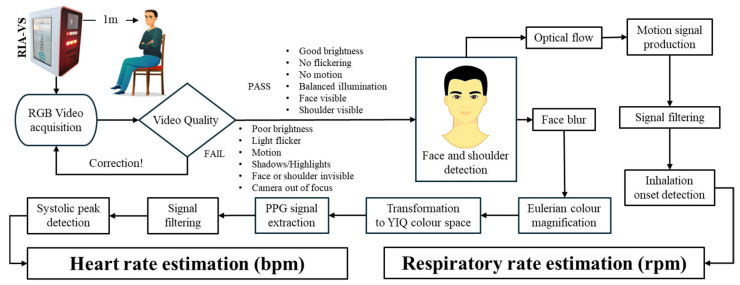
Workflow of the system for remote investigation and assessment of vital signs (RIA-VS) [28]. A camera records a facial video from ~1 m distance. The video is processed to detect and track the face and shoulders, and subtle color and motion variations are analyzed to estimate HR and RR.

The system architecture, illustrated in Figure 1, is a video-based monitoring solution comprising a high-speed camera for data acquisition and a processing unit for executing vital signs extraction algorithms. The setup was designed to ensure controlled and reproducible data collection, with participants seated approximately one meter from the camera to standardize image resolution and optimize face detection. The camera was positioned to capture a clear, unobstructed view of both the face and shoulder regions, enabling dual-modality processing, i.e., rPPG from the face rectangle, which serves as the ROI, and motion-based respiratory analysis from the shoulders. Videos that failed predefined quality criteria, including excessive motion, poor focus, or low illumination, were excluded to guarantee reliable HR and RR estimation. The video stream serves as input to a sequential processing pipeline, beginning with robust localization of the ROI. The system continuously monitors for face presence, pausing or prompting the participant if detection fails. Once the face is detected, the face rectangle is extracted for rPPG analysis, while the shoulder region is simultaneously tracked for respiratory motion. The camera and processing unit were mounted in the examination room such that the system could be used conveniently during routine consultations without altering the standard clinical workflow.

HR estimation leverages subtle, periodic skin color changes associated with blood flow. To reduce high-frequency noise and variations from slight focus differences, a Gaussian blur is applied to the facial ROI. EVM then temporally amplifies cardiac-related color changes. The RGB signals are converted to the YIQ color space, discarding luminance to isolate the chrominance channels, where the pulse signal is more robust. A composite PPG waveform is extracted, smoothed, and analyzed for systolic peaks, with inter-beat intervals averaged to yield HR in bpm. RR is derived from shoulder motion. Optical flow quantifies frame-to-frame displacement, generating a respiratory waveform. Inhalation onsets are detected, and inter-breath intervals are averaged to produce the final RR in rpm. This dual-modality framework enables accurate, simultaneous extraction of HR and RR from a single video stream under control conditions. Algorithmic pseudocode and parameter settings are provided in Appendix A.

### 2.3. Data Acquisition

The participants in this study were ambulatory adult patients (≥18 years) attending either scheduled or acute visits, able to sit upright for the duration of the video recording, and able to cooperate with standard vital sign measurements. Patients requiring immediate emergency interventions (e.g., severe respiratory distress or hemodynamic instability) were not enrolled, so the present cohort primarily reflects clinically stable patients in a supervised primary care setting. The clinical research team aims to recruit 860 subjects and perform two measurements for each, yielding nearly 2000 vital signs recordings.

Data collection began in March 2022 at a primary healthcare center in Sweden and was subsequently expanded to five primary healthcare centers. An independent clinical research team collected data in examination rooms under typical ambient indoor lighting conditions, avoiding visible light-source flickering and maintaining room temperature within 10–30 °C as specified in the clinical protocol. For each participant, two videos were recorded using a high-speed RGB camera (Basler Med Ace, Basler AG, Ahrensburg, Germany) equipped with an IMX174 CMOS sensor (Sony Corporation, Tokyo, Japan) at 390 frames per second (*f_s_*) and a frame resolution of 640 × 480 pixels. Participants were seated approximately one meter from the camera, with the face and upper torso in view. Motion artifacts were carefully controlled during recording.

Reference measurements were performed immediately before each recording using gold-standard methods: HR was measured with an automatic upper-arm blood pressure device (HEM-907, OMRON Healthcare Europe B.V., Hoofddorp, The Netherlands) combined with a TriCUFF^®^ cuff (Pressure Group AB & Co KB, Sweden). RR was measured by manually counting breaths for 60 s using a timer, in line with current clinical recommendations. These measurements represent the clinical reference methods specified in the clinical investigation plan.

In this ongoing clinical trial, 400 facial videos were collected from 200 participants, with two recordings per individual, by the clinical research team in the Västra Götaland Region, Sweden. A total of 242 adult participants (≥18 years; 105 males, 137 females; mean age 61.71 years, mean height 171.04 cm, mean weight 78.49 kg) were initially enrolled, with skin pigmentation ranging from Fitzpatrick skin types 1–6 [18]. Data collection aimed to obtain at least 200 usable recordings to account for potential technical malfunctions. Recordings from 42 participants at a center enrolling participants with Fitzpatrick skin types 5 and 6 were unusable due to technical issues in the recording room, leaving only types 1–4 for analysis.

The first 50 participants contributed 100 videos of 10 s duration, while the remaining 150 participants provided 300 videos of 15 s to enhance RR estimation and assess whether longer recordings improved other vital signs measurements without imposing substantial computational overhead. Thirteen videos were excluded due to incorrectly captured clinical reference values, resulting in 387 usable recordings (289 of 15 s and 98 of 10 s). To enable consistent comparison across measurement durations, each 15 s video was truncated to 10 s, yielding a dataset of 387 samples of 10 s each in length. These 10 s recordings were further cropped to 5 s, producing an additional 5 s dataset for evaluation of duration-dependent performance. Thus, the final dataset consisted of 289 videos of 15 s, 387 videos of 10 s, and 387 videos of 5 s in length.

Video recordings were subsequently screened for technical quality and usability (Section 2.4). After applying the predefined exclusion criteria for illumination, blur, face visibility, and motion, 295 out of 387 10 s recordings were retained for HR estimation, including 170 recordings from female participants and 125 from male participants. Among these, 285 recordings were retained for RR estimation after excluding recordings in which the shoulders were not visible. For 15 s recordings, 199 videos were deemed usable for HR and 191 for RR. All analyses were performed on a home computer equipped with an Intel^®^ Core™ i7-8750H CPU (2.20 GHz).

### 2.4. Video Quality Assessment

To ensure reliable HR and RR estimation and to adhere to the pre-specified technical quality criteria in the clinical protocol, each recording was evaluated before signal processing. Video quality assessment proceeded in a staged manner, beginning with the detection of light-source flickering and insufficient illumination, followed by assessment of image sharpness, excessive facial motion, and visibility of the shoulder region. Recordings that failed any of these criteria were excluded from subsequent analysis and are accounted for in the sample counts described in the Data acquisition Section 2.3. The following sections detail these criteria, beginning with the assessment of light source flickering.

#### 2.4.1. Flicker Detection

To ensure reliable HR and RR estimation, we implemented a video flicker detection step to identify unstable illumination that could distort rPPG and motion signals. Flicker arises from the beat frequency between artificial light sources and the camera frame rate, which can overlap with the cardiac frequency and compromise signal fidelity. For each video, consecutive grayscale frames sampled at 1 *f_s_* were compared by computing the mean absolute pixel intensity difference. A flicker event was registered when this difference exceeded an experimentally determined threshold of 2, indicating periodic luminance fluctuations. The total number of flicker events was accumulated for each recording, and videos exhibiting excessive flicker were excluded from subsequent analysis. This preprocessing step ensures uniform illumination and preserves the integrity of HR and RR signals. The critical influence of dynamic illumination on video-based vital signs extraction has been documented in recent studies [29,30], underscoring the necessity of flicker screening in clinical datasets.

#### 2.4.2. Blur Detection

To ensure sufficient image sharpness for reliable vital signs extraction, we implemented a blur-detection step based on the variance calculated after applying the Laplacian operator. The first frame of each video was converted to grayscale and filtered with a Laplacian kernel to emphasize edges and fine spatial detail. We then computed the variance of the filtered image. A high variance indicates a sharp, in focus frame with abundant edges, while a low variance corresponds to a blurred or defocused frame. Frames with variance below a predefined threshold of 4 were classified as blurry. Any video whose first frame failed this check was excluded from further analysis. The use of the Laplacian-variance method for automated focus assessment is well-established in computer vision and image-quality literature [31].

#### 2.4.3. Low Illumination

To ensure adequate lighting for reliable rPPG and motion-based vital-sign extraction, we implemented a low-illumination screening step. The first frame of each video was converted to grayscale, and the mean pixel intensity was computed. Frames with a mean grayscale value below 80 (on the 0–255 scale) were classified as poorly illuminated, indicating insufficient lighting for stable rPPG or motion signal extraction. Videos failing this criterion were excluded from further analysis. This empirical threshold was chosen based on preliminary calibration and observation that lower values corresponded to visually dark, low-contrast frames that consistently yielded degraded SNR. Previous studies confirm that inadequate ambient lighting severely reduces rPPG accuracy and may impair face detection or tracking under low-light conditions [30].

#### 2.4.4. Face Motion

Excessive face motion was assessed during the face detection step discussed in Section 2.5.1, using a combination of Kanade-Lucas-Tomasi point tracking and geometric transformation analysis [32]. Initially, feature points within the detected face bounding box were identified and tracked across consecutive video frames using the tracker. For each frame, the geometric transformation between the previously tracked points and the current frame points was computed to estimate the displacement of the face region. The center of the face bounding box was calculated for both the previous and current frames, and the Euclidean distance between these centers was used as a metric of face movement. Movements exceeding an experimental threshold value of 10 pixels were classified as excessive, triggering an error flag and exclusion of the corresponding video segment from further processing. This approach ensured that subtle head movements, which could introduce motion artifacts in both rPPG and motion signals, were reliably identified and handled, maintaining the accuracy and robustness of the vital signs’ extraction pipeline.

#### 2.4.5. Shoulder Detection

Shoulder detection was performed to enable motion-based RR estimation. The algorithm analyzed the lower quarter of the video frame, where the left and right shoulder regions were expected to appear. For each side, the prescribed rectangular region was extracted and converted to grayscale, and a Sobel edge operator was applied to highlight the top contour of the shoulder. The edge image was flipped vertically so that the topmost shoulder edge in the original image becomes the first positive row in each column; a vectorized scan then finds the first edge pixel in every column, producing a set of edge coordinates that represent the shoulder contour across the region. Columns without detected edges are discarded, and at least two valid edge points are required to proceed. A straight line was fitted to the remaining top-edge points using linear regression (first-degree polynomial). The slope of this fitted line was converted to an angle (degrees) via the inverse tangent, providing a per-side shoulder angle relative to the horizontal. In the implementation, a shoulder is considered visible if the absolute value of this angle is below 45°, a threshold chosen to reject spurious, near-vertical contours while accepting the typical shallow slope of a shoulder contour.

When both left and right shoulders are detected, an additional symmetry test is applied to increase confidence in torso visibility. The algorithm compares the left and right shoulder angles: if both angles satisfy the individual visibility criterion (|angle| < 45°) and the absolute difference between them is small (e.g., <10°), the two contours are treated as approximately symmetric and form the apexed geometry expected for an isosceles triangle with the neck at the apex. This isosceles-triangle check provides a robust indicator that both shoulders are present and approximately level relative to the camera, improving the reliability of subsequent optical flow-based respiratory motion extraction. If either side fails these checks, including insufficient edge points, an angle outside the allowed range, or a large inter-side angle difference, the shoulder is declared invisible for that side, and the frame is flagged accordingly. By detecting the shoulders in this manner, the system could reliably segment upper-torso motion and generate a clean time-series signal reflecting breathing-related movements, even under slight participant displacement or minor background clutter.

### 2.5. Heart Rate Estimation

After verifying that each recording satisfied the required environmental and quality criteria, the first preprocessing step for HR estimation involved extracting the facial region in every frame. Accurate face localization ensured a consistent ROI, providing a stable and standardized input for subsequent physiological signal analysis.

#### 2.5.1. Face Detection

Facial regions were then extracted using a multi-stage detection and tracking framework. For each video, an initial frame $I_{1}$ was used to verify the presence of a face. Candidate’s face bounding boxes $B=\left\{b_{1},b_{2},\ldots ,b_{n}\right\}$ were obtained using a Viola-Jones cascade classifier [33]. To eliminate false detections, the bounding box with the largest area was selected using Equation (1),

(1) $$b*=arg\;\underset{b_{i}\in B}{max}\;A_{i},{\;\;\;\;\;\;A}_{i}=w_{i}\cdot h_{i}$$

where $w_{i}$ and $h_{i}$ denote the width and height of the *i*^th^ candidate. Videos without a valid face detection were excluded.

After initial detection, the Kanade-Lucas-Tomasi point tracker [32] was used to propagate facial features between frames. Let ${\left\{x_{k}^{\left(t\right)}\right\}}_{k=1}^{N}$ denote the tracked feature points in frame t. The similarity transforms T between frames t and t+1 was estimated as

(2) $$x_{k}^{\left(t+1\right)}\approx Tx_{k}^{\left(t\right)},\;\;T=\left[\begin{array}{cc} sR & t\\ 0^{⊤} & 1 \end{array}\right]$$

where s is scale, R is a 2D rotation matrix, and t is translation. Bounding-box corner points $p_{i}^{\left(t\right)}$ were updated as

(3) $$p_{i}^{\left(t+1\right)}=Tp_{i}^{\left(t\right)}$$

Sudden face movements were quantified using the Euclidean distance $d_{t}$ between consecutive bounding-box centres $c^{\left(t\right)}$ and $c^{\left(t+1\right)}$ in frame t and t+1:

(4) $$d_{t}=∥c^{\left(t+1\right)}-c^{\left(t\right)}∥,{\;\;\;\;\;\;\;\;\;\;\;c}^{\left(t\right)}=\frac{1}{4}\sum_{i=1}^{4}p_{i}^{\left(t\right)}$$

Recordings were discarded if $d_{t}>10$ pixels to ensure stable tracking. Next, for each frame, a standardized facial patch of 220×220 pixels was extracted around the bounding-box center $\left(x_{c},y_{c}\right)$:

(5) $$I_{t}^{\mathrm{face}}=I_{t}\left[x_{c}-r:x_{c}+r,y_{c}-r:y_{c}+r\right],\;\;\;\;\;r=110$$

**Figure 2:**
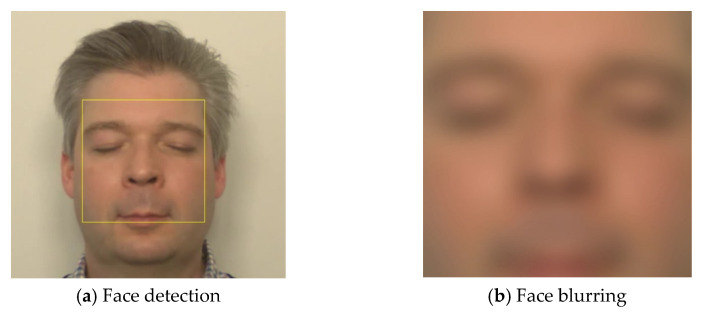
Face image processing of a representative video sample. The left panel (**a**) shows the detected face, and the right panel (**b**) shows the blurred face region used for HR estimation.

To reduce high-frequency artifacts such as wrinkles or illumination variations, Gaussian smoothing was applied:

(6) $$I_{t}^{\mathrm{smooth}}=I_{t}^{\mathrm{face}}*G_{\sigma =8}$$

where $G_{\sigma }$ is a Gaussian kernel with standard deviation σ=8, and * denotes convolution. Figure 2 shows the original face image obtained using Equation (1) alongside the blurred output produced using Equation (6).

This procedure produced a sequence of temporally aligned, normalized facial images, which were used as input for subsequent physiological signal extraction, including EVM and signal analysis for HR estimation.

#### 2.5.2. Eulerian Video Magnification

EVM was employed to enhance subtle pulsatile variations in the facial region to facilitate non-contact HR estimation. This method amplifies small, periodic changes in pixel color caused by cardiac activity while suppressing large-scale motion and lighting fluctuations that could obscure the pulse signal [11]. The resulting magnified frames provide a visually interpretable representation of the systolic and diastolic phases of the cardiac cycle. The human HR typically ranges from 50 to 200 bpm, corresponding to a temporal frequency band of approximately $f_{\mathrm{low}}=\frac{50}{60}\approx 0.83\;Hz$ and $f_{\mathrm{high}}=\frac{200}{60}\approx 3.33\;Hz$. Given the high frame rate of the camera ($f_{s}=390\;Hz$), this frequency range defines the passband for the temporal band-pass filter applied to the color time series at each pixel. By restricting processing to this physiologically relevant range, irrelevant low-frequency movements and high-frequency noise are largely suppressed, improving the reliability of the cardiac signal.

To perform the EVM, each pre-processed facial frame $I_{t}(x,y)$ was first converted from RGB to NTSC color space, separating luminance and chrominance components to facilitate color-based amplification of subtle pulsatile changes. The frames were then decomposed into a six-level Laplacian pyramid, which separates spatial frequency bands to isolate regions where pulse-related intensity variations are most pronounced. The Laplacian coefficient at pyramid level l is defined in Equation (7) as

(7) $$L_{l}(t)={\mathrm{LaplacianLevel}}_{l}(I_{t})$$

where $L_{l}(t)$ isolates spatial structures at scale l while suppressing larger-scale facial movements. This decomposition ensures that amplification primarily targets subtle variations due to cardiac activity rather than larger facial motions.

Now, for each pyramid level l, the temporal sequence of each pixel, $s_{l}(t)$, was extracted and passed through a band-pass filter h(t) that retains only frequencies within the cardiac range

(8) $${\overset{\sim }{s}}_{l}(t)=(s_{l}*h)(t)$$

where ∗ denotes convolution along the temporal axis. The filtered signal was then amplified using a level-dependent factor $\alpha_{l}$ (see Appendix A, Step 5 for computing $\alpha_{l}$),

(9) s^l(t)=sl(t)+αls~l(t)

**Figure 3:**
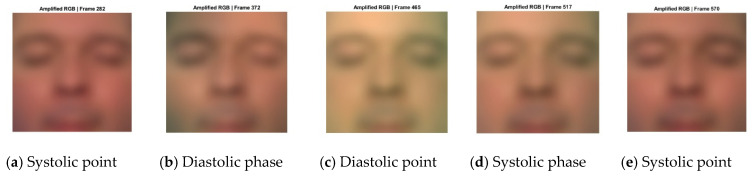
Sequence of color-magnified facial images from a representative video illustrating cyclic changes in facial chromaticity across the cardiac cycle. The frames highlight the (**a**) systolic point, (**b**) diastolic phase, (**c**) diastolic point, (**d**) systolic phase, and return to the (**e**) systolic point. These amplified variations visualize cardiac pulsatility and support subsequent non-contact HR estimation.

The amplified pyramid levels L^l(t) were collapsed through inverse Laplacian reconstruction to generate the magnified frames I^t(x,y). These magnified frames reveal the cyclical modulation of facial skin color driven by the cardiac cycle. As shown in Figure 3, the enhanced sequence makes it possible to observe the progression of pulsatile hemodynamics across successive frames. The illustrated cycle begins at a systolic point, transitions into the diastolic phase, reaches the diastolic point, and then returns through the systolic phase to a subsequent systolic point. This periodic color modulation underpins the extraction of a non-contact HR signal and demonstrates the utility of color-based Eulerian magnification for visualizing cardiac-induced facial skin changes.

#### 2.5.3. Color Transformation and rPPG Signal Extraction

**Figure 4:**
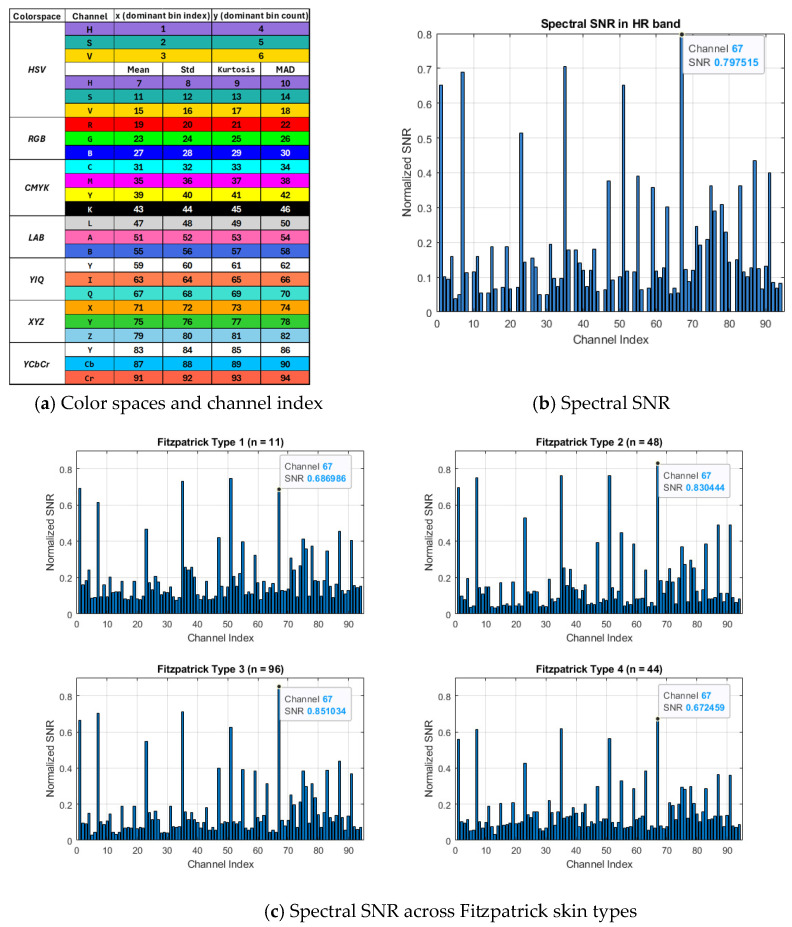
Performance of color channels for rPPG. (**a**) Channel indices derived from multiple color spaces, including HSV, RGB, CMYK, LAB, YIQ, XYZ, and YCbCr. (**b**) Normalized spectral SNR in the HR band for rPPG signals from each channel derived from 15 s videos. The Q channel (channel 67, *n* = 199 samples) exhibits the highest SNR, approaching 0.8, indicating a highly periodic and robust pulsatile signal. (**c**) Stratification by Fitzpatrick skin types 1–4 shows that the Q channel consistently maintains superior SNR across all groups (0.67–0.85), confirming its reliability and suitability for rPPG extraction across diverse skin tones.

Before rPPG extraction, raw signals were extracted from each chrominance channel of the color-magnified face images derived from 15 s videos across multiple color spaces (HSV, RGB, CMYK, LAB, YIQ, XYZ, and YCbCr) and evaluated for cardiac periodicity using dominant bin analysis and normalized spectral SNR. Among all channels, the Q channel from the YIQ color space (channel 67, *n* = 199) consistently exhibited the highest SNR, approaching 0.8 on the normalized 0–1 scale, indicating a robust and periodic pulsatile signal suitable for reliable HR estimation. To confirm the stability of this selection across diverse populations, the spectral SNR was further analyzed by skin type using the Fitzpatrick scale (Figure 4c). The Q channel consistently maintained superior SNR across all Fitzpatrick Types 1–4 (0.67–0.85), demonstrating that it reliably captures the underlying pulsatile signal regardless of skin tone. This stability reduces the need for extensive post-processing, minimizes computational overhead, and validates the Q channel as the optimal source for rPPG extraction in heterogeneous populations.

**Figure 5:**
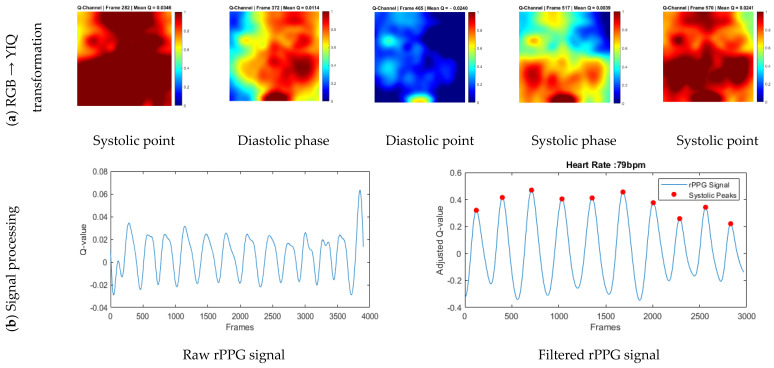
rPPG signal extraction and HR estimation using the Q channel. (**a**) Normalized false-color maps of the Q-channel illustrating five representative cardiac phases across the face region. (**b**) Corresponding rPPG signal computed as the temporal mean of the Q-channel, showing both the raw and filtered signals. Detected systolic peaks are indicated and were used to calculate the HR displayed as 79 bpm.

A raw rPPG signal was then generated by computing the temporal mean of the Q-channel across the face region for each frame, capturing beat-to-beat blood-volume variations for HR estimation (Figure 5b). To illustrate the cardiac cycle, five representative frames at the systolic point, diastolic phase, diastolic point, systolic phase, and systolic point were extracted and visualized as amplified RGB images and normalized false-color Q-channel maps, with the temporal mean displayed alongside (Figure 5a).

#### 2.5.4. rPPG Signal Filtering

The raw Q-channel rPPG signal was pre-processed to remove noise and baseline drift before HR estimation. Outliers were first removed by clipping the signal to the 2nd and 98th percentiles using Equation (10):

(10) $$x_{\mathrm{clip}}[n]=\{\begin{array}{cc} P_{2}, & \;\;\;\;\;\;\;\;\;\;\;\;\;\;x[n]<P_{2}\\ x[n], & {\;\;\;\;\;\;P}_{2}\leq x[n]\leq P_{98}\\ P_{98}, & \;\;\;\;\;\;x[n]>P_{98} \end{array}$$

A moving-average filter was then applied to smooth short-term fluctuations:

(11) $$x_{\mathrm{MA}}\left[n\right]=\frac{1}{L_{1}}\sum_{k=0}^{L_{1}-1}x_{\mathrm{clip}}\left[n-k\right],\;\;\;\;\;\;\;\;L_{1}=\frac{f_{s}}{4}$$

This was followed by Gaussian smoothing to further reduce noise:

(12) $$x_{\mathrm{smooth}}\left[n\right]=\sum_{k=-\left\lfloor \frac{L_{2}}{2}\right\rfloor }^{\left\lfloor \frac{L_{2}}{2}\right\rfloor }x_{\mathrm{MA}}\left[n-k\right]\cdot g\left[k\right],\;\;g[k]=\frac{1}{\sqrt{2\pi }\sigma }exp(-\frac{k^{2}}{2\sigma^{2}})$$

where $L_{2}=f_{s}/2\;$and $\sigma =L_{2}/6$.

The processed signal was then confined between the first and last valleys, defined as the local minima of $x_{\mathrm{smooth}}[n]$, to segment a complete cardiac cycle using Equation (13):

(13) $$x_{\mathrm{seg}}\left[n\right]=x_{smooth}\left[n\right],\;\;n\in [n_{\mathrm{valley}}^{\left(1\right)},n_{\mathrm{valley}}^{\left(K\right)}]$$

#### 2.5.5. Final Output

Systolic peaks were detected as local maxima within this segment. Finally, the HR was calculated from the number of detected peaks and the segment duration T using Equation (14):

(14) $$\mathrm{HR}=\frac{N_{\mathrm{peaks}}}{T}\times 60,T=\frac{n_{\mathrm{valley}}^{\left(K\right)}-n_{\mathrm{valley}}^{\left(1\right)}}{f_{s}}$$

where $N_{\mathrm{peaks}}$ is the number of detected systolic peaks, and T is the duration of the analyzed segment in seconds. The resulting filtered rPPG signal, shown in Figure 5b, is smooth and free of outliers, with detected systolic peaks used to estimate an HR of 79 bpm, demonstrating that the Q channel reliably captures both temporal and spatial pulsatile dynamics for non-contact HR estimation.

### 2.6. Respiratory Rate Estimation

Respiration was estimated from the subtle superior-inferior displacement of the shoulders, extracted concurrently with facial-image acquisition. Shoulder regions were identified as described in Section 2.4.5 using anatomically guided rectangular windows positioned in the lower quarter of the frame. Edge-based contour extraction and linear regression were applied to derive shoulder orientation, while symmetry constraints ensured adequate torso visibility.

For each frame $I_{t}$, a grayscale representation was generated to stabilize motion estimation, and dense optical flow was computed between consecutive frames $\left(I_{t},I_{t+1}\right)\;$using the variational method [34]. The resulting two-dimensional flow field $v_{t}(x,y)\;$is given in Equation (15), which encodes the frame-to-frame displacement at each pixel (x,y), where $u_{t}\left(x,y\right)\;$and $v_{t}\left(x,y\right)$ represent the horizontal and vertical components of motion, respectively.

(15) $$v_{t}(x,y)=(u_{t}(x,y),{\;v}_{t}(x,y))$$

Because respiration induces predominantly vertical displacement of the shoulders, only the vertical component $v_{t}(x,y)$ was retained. A shoulder-motion trace was then constructed by spatially averaging across the detected shoulder region Ω using Equation (16):

(16) $$s(t)=\frac{1}{|\Omega |}\sum_{(x,y)\in \Omega }v_{t}(x,y)$$

To suppress noise and artifacts, extreme deviations in s(t) were replaced through linear interpolation using a median–absolute–deviation threshold, followed by smoothing via convolution with a Gaussian kernel h(t) using Equation (17):

(17) $$\overset{\sim }{s}(t)=s(t)*h(t)$$

The filtered signal $\overset{\sim }{s}(t)$ exhibits a quasi-periodic oscillatory pattern corresponding to upward motion representing inhalation, and downward motion representing exhalation. Respiratory cycles were identified by detecting successive local maxima in $\overset{\sim }{s}(t)$. Denote the maxima as $\{t_{k}^{\max}{\}}_{k=1}^{N_{\max}}$. A respiratory cycle corresponds to the interval between consecutive maxima as given in Equation (18):

(18) $$T_{k}=t_{k+1}^{\max}-t_{k}^{\max}$$

Cycle durations shorter than 1 s or longer than 12 s were rejected as physiologically implausible. The instantaneous RR for cycle k is computed using Equation (19):

(19) $$R_{k}=\frac{60}{T_{k}}$$

The final RR estimate is the mean across all valid cycles and is computed using Equation (20):

(20) $$RR=\frac{1}{N}\sum_{k=1}^{N}R_{k}$$

**Figure 6:**
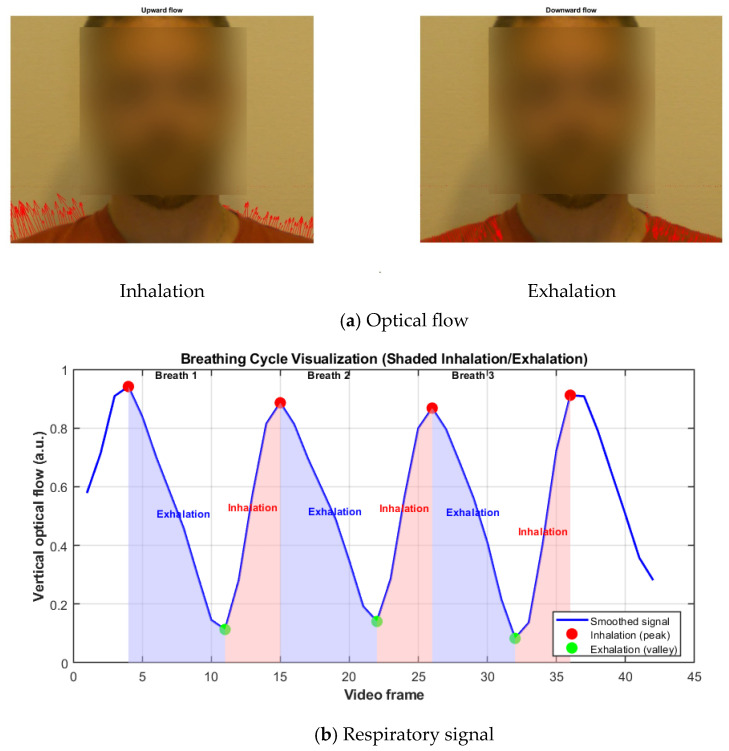
Respiratory activity: (**a**) shoulder movements during inhalation and exhalation are indicated by upward and downward arrows showing motion flow from optical flow; (**b**) the respiratory signal derived from optical flow of the shoulders, shows the smoothed trace in blue, with shaded inhalation (red) and exhalation (blue) phases labeled internally, peaks (red) and valleys (green) marking maximal inhalation and exhalation, and breaths numbered at exhalation start; video frames are stepped out for visualization.

This dual estimation of RR and individual respiratory cycles enables both average breathing frequency quantification and cycle-wise temporal analysis, allowing detection of transient irregularities and providing a more detailed physiological assessment than mean rate alone [12]. Reporting individual respiratory cycles in addition to the RR allows identification of cycle-to-cycle variations, facilitates detection of irregular or asymmetric breathing patterns, and improves robustness against transient outliers, which is particularly important in non-contact, video-based respiration monitoring. The performance of this method is illustrated in Figure 6, where panel (**a**) shows vertical shoulder movements during inhalation and exhalation indicated by upward and downward arrows, and panel (**b**) displays the smoothed respiratory signal derived from optical flow, with inhalation and exhalation phases shaded, peaks and valleys marking maximal inhalation and exhalation, and breaths numbered at exhalation start. By leveraging vertical optical-flow dynamics that are well-established for extracting subtle physiological motion, this approach enables robust, non-contact respiration measurement under typical indoor recording conditions.

### 2.7. Statistical Analysis

#### 2.7.1. Performance of the HR Estimation Algorithm

For each usable recording and each window duration (5, 10, and 15 s), HR was estimated from facial video-derived rPPG signals using the proposed processing pipeline (Equation (14)), and performance was evaluated to assess accuracy, reproducibility, reliability, and computational efficiency. The corresponding clinical reference HR values were obtained from the automatic upper-arm blood pressure device (HEM-907).

To quantify accuracy, we computed the MAE and root-mean-square error (RMSE) between estimated and reference HR across recordings at each duration. Linear regression and Pearson’s correlation coefficient r were used to assess linear association. Agreement was further evaluated using Bland–Altman analysis, plotting the difference between estimated and reference HR against their mean, and computing the mean bias and 95% LoA defined as ±1.96 standard deviation. This analysis allowed detection of potential systematic biases and identification of trends in over- or underestimation across the HR range, complementing correlation-based metrics [35].

As an additional clinically oriented performance measure, we calculated the proportion of estimates falling within ±10 bpm of the reference HR. This threshold was chosen a priori based on commonly used alert thresholds in early warning scores and on prior literature on clinically meaningful changes in HR; within this range, small differences between methods would rarely alter triage categories or management decisions in typical primary care and emergency settings [36].

HR estimation was also analyzed in terms of temporal resolution and computational efficiency. Shorter video segments, such as 5 s, enable rapid assessment and reduced processing times but are more susceptible to transient motion artifacts and higher variance in beat detection. Conversely, longer segments of 10 s to 15 s improve robustness by averaging over more cardiac cycles, thereby enhancing estimation accuracy and reproducibility, albeit at the cost of increased computation. Processing times were recorded for each measurement duration, and variability in execution was evaluated to ensure that the proposed approach is feasible for real-time monitoring without imposing excessive computational load.

By comparing performance across multiple recording durations, the analysis provides insight into the minimum acquisition time required for reliable non-contact HR monitoring on an edge device, while balancing temporal resolution and computational efficiency. This approach also enables identification of the optimal segment length for clinical applications, considering natural physiological variability, motion artifacts, and practical constraints of video acquisition. The results of the statistical analysis of HR estimation are presented in Section 3.1.

#### 2.7.2. Performance of the RR Estimation Algorithm

Unlike HR estimation, RR analysis requires attention to individual respiratory cycles, as clinical respiration assessment exhibits substantial inter-rater variability, even among trained clinicians. Discrepancies of up to ±6–8 rpm have been reported during manual counting, often due to short counting windows, subtle chest movements, and human perceptual bias [37,38,39]. To address this, we created an auxiliary ground-truth dataset based on video-derived respiratory cycles. Each recording was reviewed in slow motion, typically at one-quarter speed, to enable frame-level counting of complete inhalation-exhalation cycles. The number of visually confirmed cycles was converted to a reference respiration rate using ${\mathrm{RR}}_{video}=\frac{N_{cycles}}{T}\times 60,$ where $N_{cycles}$ is the number of observed cycles in the video, and T is the recording duration in seconds. This cycle-based reference from the slow-motion videos provides a temporally precise standard that complements the clinical RR and reduces ambiguity from irregular or subtle breathing patterns [40,41,42]. While manual clinical counting yields a single rate for the entire recording and is sensitive to window length and observer attention, video-derived annotations capture cycle-level temporal dynamics, mitigate ambiguity from irregular breathing, such as during occasional sighs, and reduce inter-rater disagreement through slow-motion validation. Combining the clinical respiratory rate ${\mathrm{RR}}_{clinic}$ with the observer-verified respiratory rate extracted from slow-motion videos ${\mathrm{RR}}_{video}$ enables a more rigorous evaluation of vision-based respiration estimation under realistic indoor conditions.

Automated RR estimates were obtained from the filtered shoulder-motion signal using Equation (20). These estimates were then compared against both $RR_{clinic}$ and $RR_{video}$. Accuracy was quantified in terms of MAE and RMSE, while correlation with the reference rates was assessed using the Pearson correlation coefficient. Additionally, the proportion of estimates within ±5 rpm was computed to reflect clinically acceptable performance standards [26].

Because natural respiratory variability may cause differences in rates across recordings of different durations, separate analyses were conducted for 10 s and 15 s video recordings. Reference values and automated estimates were evaluated for each recording type using MAE, RMSE, Pearson correlation, and the percentage of estimates within the clinically acceptable ±5 rpm range. Bland–Altman analysis [35] was further performed to quantify systematic bias and LoA between the estimated and reference RR. The results of the statistical analysis of RR estimation are presented in Section 3.2.

## 3. Results

### 3.1. Performance of HR Estimation Algorithm

**Figure 7:**
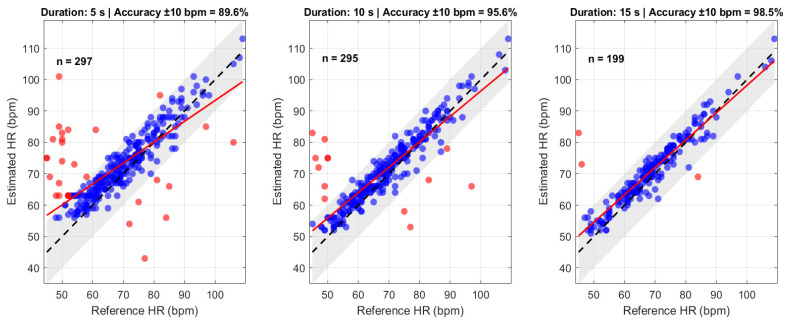
Scatter plots of HR estimation versus reference values across different measurement durations. Estimated HR values are shown for 5 s, 10 s, and 15 s windows. The dashed line represents perfect agreement (*y* = *x*), and the solid line shows the linear regression fit. The shaded area indicates the ±10 bpm acceptance band used to evaluate accuracy. Red circles indicate errors (outside ±10 bpm), and blue circles represent accurate estimates (within ±10 bpm). Accuracy improves with longer durations: 89.6% (5 s), 95.6% (10 s), and 98.5% (15 s), demonstrating increasingly reliable HR estimation with extended measurement windows.

The scatter plots in Figure 7 illustrate the HR estimation performance of the proposed non-contact method across measurement durations of 5 s, 10 s, and 15 s. Each plot compares the estimated HR obtained from the facial videos on the y-axis with the reference HR on the x-axis. The dashed black line denotes the line of unity (y = x), while the solid red line represents the linear regression fit. The shaded grey region corresponds to the ±10 bpm acceptance band used to assess estimation accuracy. A clear trend is observed, i.e., increasing the measurement duration reduces the spread of data points around the line of unity. The 5 s recording exhibits several outliers beyond the acceptance band, whereas the 10 s and 15 s show progressively tighter clustering and fewer deviations. Correspondingly, accuracy improves from 89.6% (5 s) to 95.6% (10 s) and 98.5% (15 s). The quantitative metrics further confirm this improvement. The 5 s window results in an MAE of 5.63 bpm and an RMSE of 8.96 bpm, with a correlation coefficient of r = 0.75 (*p* < 0.05). Increasing the duration to 10 s reduces the MAE to 3.81 bpm and the RMSE to 6.22 bpm, while improving correlation to r = 0.88 (*p* < 0.05). The 15 s window produces the most accurate estimates, achieving an MAE of 3.25 bpm, an RMSE of 4.88 bpm, and a correlation of r = 0.93 (*p* < 0.05).

**Figure 8:**
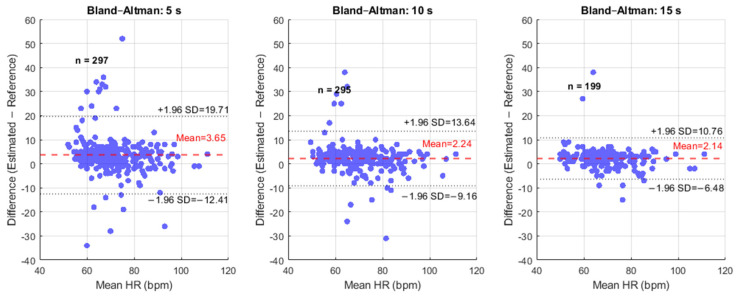
Bland–Altman analysis of HR estimation across measurement durations. The difference between estimated and reference HR is plotted against their mean for 5 s, 10 s, and 15 s recordings; mean bias decreases from 3.65 bpm (5 s) to 2.24 bpm (10 s) and 2.14 bpm (15 s), while LoA narrow from −12.41 to +19.71 bpm, −9.16 to +13.64 bpm, and −6.48 to +10.76 bpm, respectively, indicating progressively improved accuracy and precision with longer measurement windows.

The Bland–Altman plots in Figure 8 assess the agreement between the reference and estimated HR values for the 5 s, 10 s, and 15 s recordings. Each plot displays the difference between the estimated and reference HR on the y-axis against their mean on the x-axis, enabling visual identification of systematic deviations and potential dependence of errors on HR magnitude. The mean bias shown as the red dashed line represents the average difference between methods and indicates the degree of systematic over- or underestimation by the non-contact algorithm. The LoA, shown as grey dashed lines and defined as the mean bias ±1.96 × SD of the differences, quantifies the range within which 95% of paired measurement differences are expected to fall, thereby characterizing the precision and random variability of the estimates.

Analysis of the 5 s recording reveals a mean bias of 3.65 bpm, the highest of the three durations, indicating the largest systematic overestimation. The LoA spans from −12.41 bpm to +19.71 bpm, highlighting substantial random error, with some differences exceeding 20 bpm. Increasing the duration to 10 s reduces the mean bias to 2.24 bpm and narrows the LoA to −9.16 bpm to +13.64 bpm. This reduction in both systematic and random error demonstrates that longer windows effectively average transient fluctuations, improving precision. For 15 s recordings, the mean bias is further minimized to 2.14 bpm, with the LoA spanning −6.48 bpm to +10.76 bpm, representing the narrowest range of differences and the highest level of agreement. The tighter clustering of data points within this band confirms that the 15 s duration provides the most consistent and reliable HR estimates.

**Figure 9:**
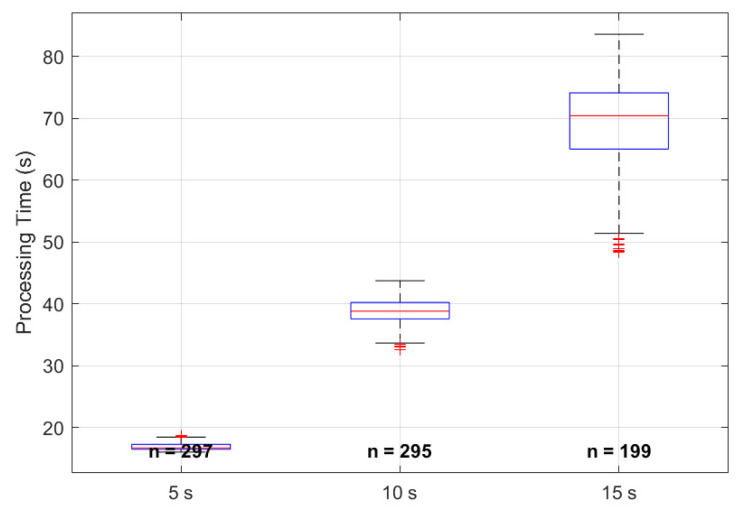
Processing time of the non-contact HR estimation algorithm across measurement durations. Box plots show processing time distributions for 5 s, 10 s, and 15 s recordings. Median times increase from 16.9 s (IQR 15.5–17.5 s) to 38.8 s (IQR 37.0–40.0 s) and 68.4 s (IQR 66.0–74.0 s), with occasional outliers above 80 s for the longest recordings, illustrating the trade-off between computational cost and HR estimation precision.

The box plots presented in Figure 9 illustrate the execution speed of the non-contact HR estimation algorithm, measured as processing time, across the 5 s, 10 s, and 15 s measurement windows. This analysis provides insight into the practical feasibility of the system and its ability to operate in near real-time. Each box plot summarizes the distribution of processing times for the respective recording duration. The central red line represents the median, the blue box spans the interquartile range (IQR, 25th to 75th percentiles), the dashed whiskers extend to the minimum and maximum non-outlier values, and red plus signs denote outliers.

The results reveal a near-linear increase in processing time with longer measurement durations, highlighting the computational trade-off between improved accuracy and increased processing requirements. For the 5 s window, the median processing time is 16.9 s, with a narrow IQR (15.5–17.5 s) and short whiskers, indicating highly consistent performance. This fast execution is advantageous for applications requiring rapid feedback, although accuracy and precision are comparatively lower at this duration. Increasing the window to 10 s more than doubles the median processing time to 38.8 s, with an IQR of approximately 37.0 to 40.0 s, reflecting the additional computational load from processing a larger volume of video data while maintaining relatively low variability.

The 15 s window, which produced the highest accuracy (98.5%) and the narrowest LoA in the HR estimation analysis, requires the longest processing time on an edge device, such as a home computer in this case, with a median of 68.4 s and a wider IQR (66.0 to 74.0 s). Whiskers extend beyond 80 s in some cases, reflecting increased variability due to factors such as memory allocation, CPU load for larger video segments, and fluctuations in system resource availability during computation.

### 3.2. Performance of RR Estimation Algorithm

**Figure 10:**
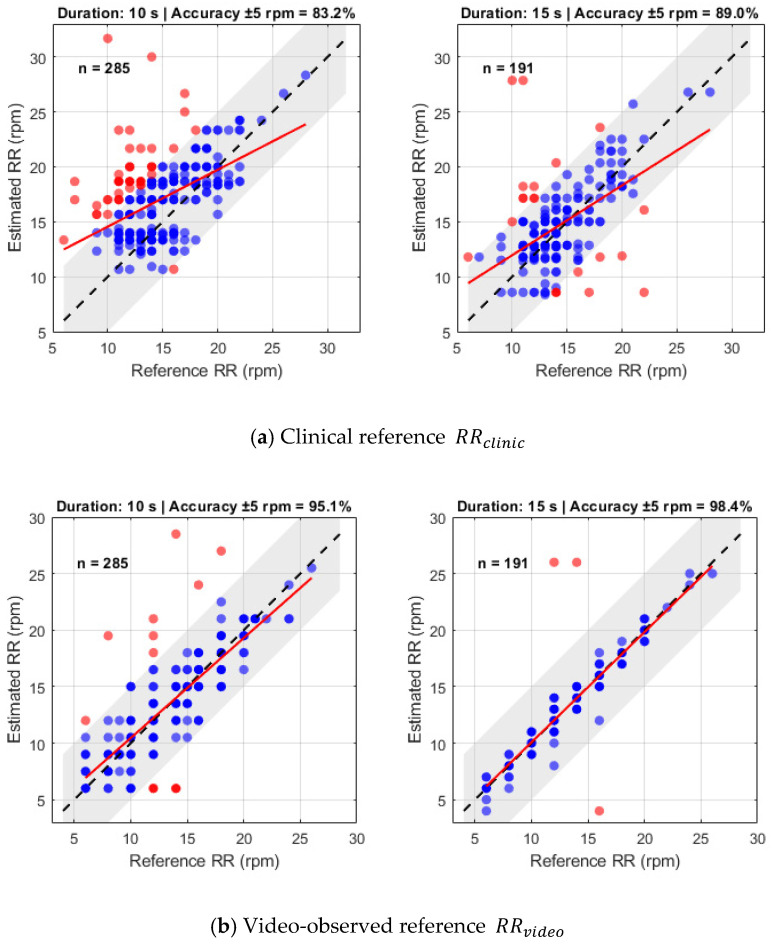
Scatter plots comparing estimated RR with (**a**) clinical reference $RR_{clinic}$ and (**b**) video-observed $RR_{video}$ for 10 s and 15 s video recordings. The ±5 rpm acceptance band, unity, and regression lines are shown. Red circles depict errors (outside ±5 rpm), and blue circles represent accurate estimates (within ±5 rpm). Estimates from 15 s videos exhibit tighter clustering and stronger alignment with $RR_{video}$ highlighting algorithm robustness.

The performance of RR estimation from the filtered shoulder-motion signal was assessed against two ground-truth standards $RR_{clinic}$ and $RR_{video}$. Analyses were conducted for both 10 s and 15 s videos to examine the effect of measurement duration on accuracy and reliability. Scatter plots with a clinically acceptable ±5 rpm tolerance band (Figure 10) showed clear differences across durations and reference types. Increasing the recording length improved the agreement between estimated and reference RR values. Additionally, the choice of reference had a marked impact on apparent performance: $RR_{clinic}$ displayed greater dispersion, consistent with the known variability of manual counting, whereas $RR_{video}$ exhibited tight clustering around the line of identity, indicating high-fidelity agreement with the proposed method.

When compared against $RR_{clinic}$, the algorithm achieved an accuracy of 83.2% for 10 s videos, improving to 89.0% for 15 s. Despite this improvement, the data showed some scatter and the linear regression line tended to fall slightly below the line of unity, suggesting a modest systematic underestimation consistent with the variability reported in manual clinical counting. In contrast, validation against $RR_{video}$, which provides temporally precise cycle-level annotations, highlighted the intrinsic capability of the method. Accuracy reached 95.1% for 10 s videos and 98.4% for 15 s, with the data forming a near-perfect alignment with the line of unity. This result demonstrates the robustness of shoulder-motion-based RR estimation when assessed against a high-fidelity, cycle-resolved reference.

Quantitative analysis confirmed these trends. For $RR_{clinic}$, MAE decreased from 3.08 rpm for 10 s recordings to 2.41 rpm for 15 s recordings. When compared with $RR_{video}$, MAE reached a minimum of 0.69 rpm for 15 s recordings, representing over 70% error reduction relative to clinical reference. RMSE and Pearson correlation further supported these results, with correlation rising from r = 0.50 for 10 s recordings to r = 0.54 for 15 s and reaching r = 0.90 for 15 s recordings against $RR_{video}$ (*p* < 0.05). These findings demonstrate strong linear agreement and high predictive accuracy of the RR estimation algorithm when validated against video-observed ground truth.

**Figure 11:**
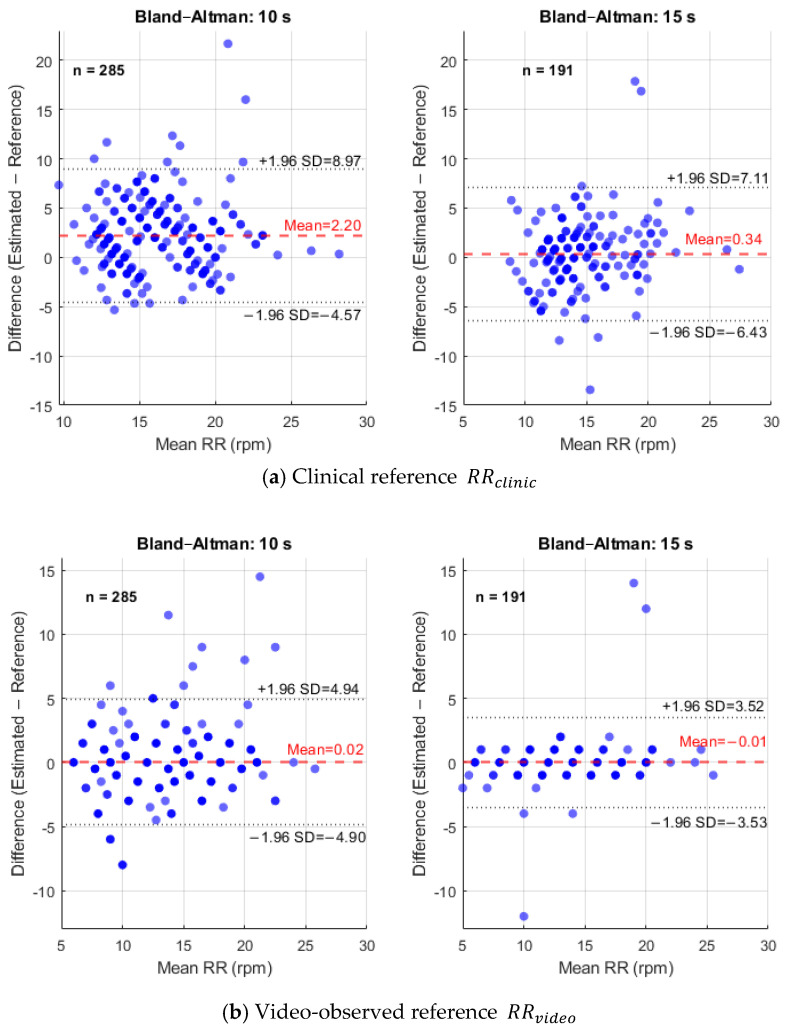
Bland–Altman plots comparing estimated RR with (**a**) the clinical reference $RR_{clinic}$ and (**b**) the video-observed reference $RR_{video}$ for 10 s and 15 s recordings. Solid lines indicate the mean bias, and dashed lines denote the 95% LoA. The clinical comparison using $RR_{clinic}$ shows larger bias and wider limits, reflecting manual assessment variability, whereas the video-observed reference $RR_{video}$ exhibits near-zero bias and narrow limits, demonstrating high precision and strong agreement with the automated method.

Bland–Altman analysis quantified agreement between the estimated RR and the two reference standards, assessing both systematic bias and precision across the RR range. Compared with $RR_{clinic}$, the plots (Figure 11a) showed larger systematic and random errors, reflecting the inherent variability of manual respiratory measurements. The mean bias was positive for both durations, indicating that the clinical reference tended to report higher rates than the automated estimates. This bias decreased with longer recordings, from 2.20 rpm for 10 s to 0.34 rpm for 15 s, suggesting that shorter recordings amplify discrepancies from manual counting. The LoA narrowed from +8.97 and −4.57 rpm for 10 s, to +7.11 and −6.43 rpm for 15 s. Despite improved precision with longer recordings, the overall range of ~13.5 rpm highlights that much of the dispersion arises from the clinical reference itself rather than the RR estimation algorithm.

In contrast, Bland–Altman analysis against the video-observed reference $RR_{video}$ (Figure 11b) revealed excellent agreement, highlighting the intrinsic precision of the shoulder-motion method. The mean bias was effectively zero, 0.02 rpm for 10 s and −0.01 rpm for 15 s, confirming negligible systematic error. Precision improved with duration: the LoA narrowed from +4.94 to −4.90 rpm for 10 s recordings to +3.52 to −3.53 rpm for 15 s. The tight clustering around zero in the 15 s plot demonstrates high reliability, with over 95% of measurements within a narrow, clinically acceptable range.

**Figure 12:**
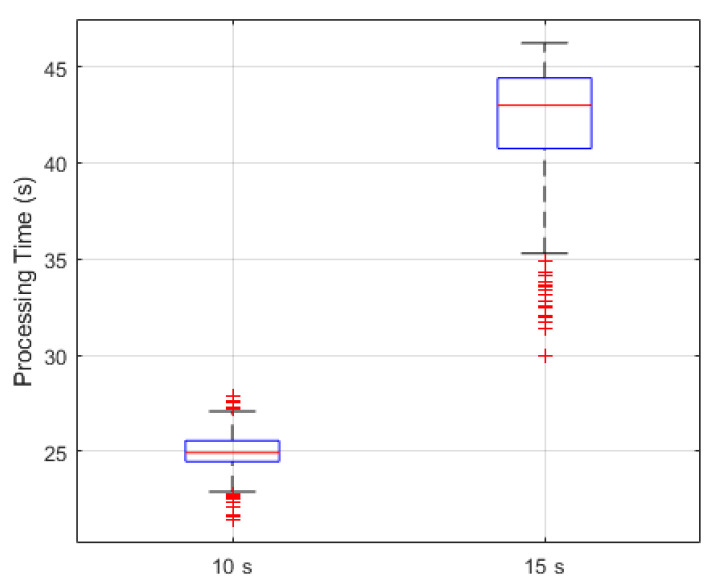
Processing time. Box plots of video assessment computation time for 10 s and 15 s durations, showing increased processing for higher accuracy at 15 s. Boxes: 25th-75th percentile (IQR); whiskers: min-max; outliers: red plus signs.

The computational efficiency of the RR estimation framework was examined to assess its suitability for practical deployment. Figure 12 summarizes the algorithm’s execution characteristics for the 10 s and 15 s recordings. The 10 s recordings exhibit a median execution of approximately 25 s with a narrow distribution (IQR ≈ 24.8–25.8 s), reflecting stable and consistent performance under shorter acquisition durations. In contrast, the 15 s recordings show a markedly broader distribution, with a median of roughly 43 s and an IQR of about 41–45 s, indicating increased computational demand and greater sensitivity to variability in video content and system load.

## 4. Discussion

We validated a dual-modality, non-contact system for simultaneous HR and RR estimation from facial videos acquired with a high-speed RGB camera. For HR estimation, the framework employs EVM to enhance subtle pulsatile color variations in the YIQ chrominance domain, specifically the Q channel, thereby increasing SNR and stabilizing rPPG extraction, while RR is derived from motion-based shoulder tracking. The use of EVM is restricted to recordings that pass stringent video-quality assessment, excluding segments affected by flicker, blur, excessive motion, poor illumination, or unstable face detection, thereby mitigating motion sensitivity during amplification. This integration of complementary modalities with strict data curation enables reliable monitoring over short measurement windows, advancing non-contact vital-sign assessment toward practical clinical and home care.

The tolerance analysis (Table 1) confirms the robustness of the proposed algorithm across segment durations. At 15 s, HR and RR estimates show strong agreement, 91.5% of HR within ±5 bpm and 98.5% within ±10 bpm, and 97.4% of $RR_{video}$ within ±3 rpm and 98.4% within ±5 rpm, indicating that short windows can yield reliable measurements with efficient processing, with average HR processing time of 68.3 s. Reduced tolerance at 10 s and 5 s reflects the trade-off between window length and estimation precision, particularly for slower respiratory signals. The consistently high accuracy at 15 s suggests that this duration provides sufficient temporal information to mitigate transient noise, stabilize frequency-domain peaks for rPPG-based HR estimation, and capture multiple respiratory cycles. Together, these findings indicate that 15 s represents a practical lower bound for rapid, accurate non-contact HR and RR monitoring, balancing acquisition speed with measurement reliability. This window length also enables rapid, non-contact vital-sign assessment without patient fatigue or prolonged exposure, making it especially suitable for scenarios requiring swift monitoring, such as COVID-19 protocols.

The use of a high-speed RGB camera at 390 *f_s_* further supports the robustness of ultra-short 15 s measurements. Compared with conventional 30–60 *f_s_* acquisition, the higher frame rate provides finer temporal resolution of subtle color changes in facial skin, improving the fidelity of rPPG signals and reducing aliasing in frequency-domain analyses. This is particularly beneficial for short recordings, where fewer frames at lower frame rates would limit peak detection and degrade SNR, potentially reducing HR and RR accuracy. Moreover, the increased frame rate enables more effective EVM without introducing motion artifacts, further enhancing the quality of the extracted pulsatile signals.

In the context of prior clinical studies, the present results demonstrate improved efficiency without compromising accuracy. Allado et al. reported that rPPG-based HR estimation over 60 s recordings achieved approximately 96.2% agreement with ECG using Bland–Altman analysis [21], while a hospital-based trial using 60 s recordings for RR estimation showed 96.0% agreement with a clinical reference [26]. By comparison, the proposed system achieves 98.5% HR accuracy (MAE: 3.25 bpm; r = 0.93) and 98.4% RR agreement (MAE: 0.69 rpm) using only 15 s recordings, with explicit processing-time reporting confirming feasibility on consumer-grade hardware. Some studies employ longer windows (30–60 s) to ensure stable signal extraction and reduce noise [43,44,45], whereas shorter windows below 30 s are generally less reliable. Notably, Verkruysse et al. [45] reported moderate SNR (~60%) for non-contact pulse extraction using ambient light and raw RGB frames, while Philips’ commercial system [46] achieves higher robustness but relies on longer acquisition windows and proprietary post-processing. In contrast, RIA-VS attains SNRs approaching 80% using only 15 s recordings by combining EVM amplification with Q-channel extraction, demonstrating both higher signal quality and greater efficiency than these prior approaches. Collectively, these findings indicate that our dual-modality system not only exceeds previously reported accuracy and precision but also achieves rapid acquisition and transparent computational performance.

**Figure 13:**
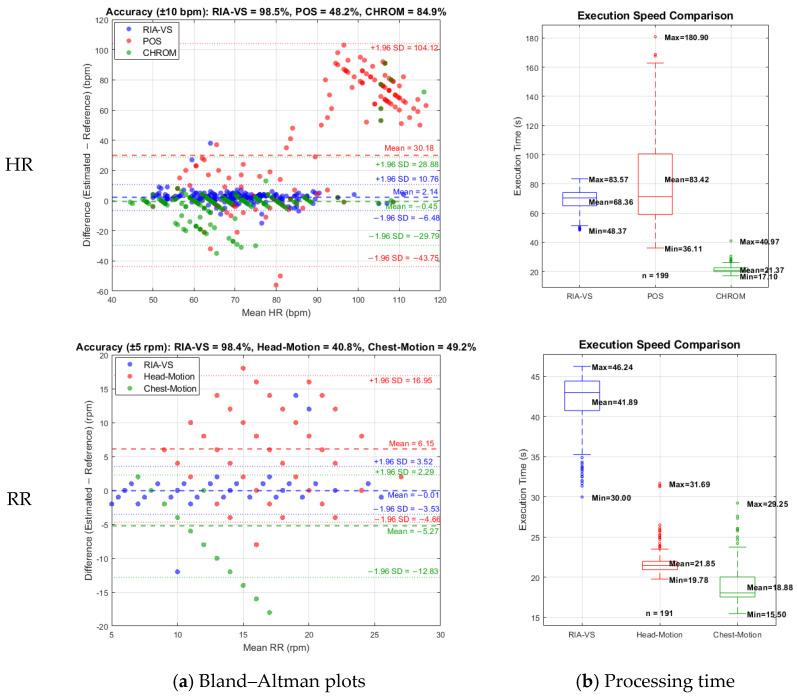
Head-to-head comparison of the proposed RIA-VS framework with recent state-of-the-art methods for HR and RR estimation. (**a**) Bland–Altman plots show agreement between estimated and reference values (*n* = 199 for HR; *n* = 191 for RR). For HR, RIA-VS demonstrates high precision (mean bias = 2.14 bpm, 98.5% accuracy), substantially outperforming the motion-robust POS method [47] (mean bias = 30.18 bpm, 48.2% accuracy) and the learning-based CHROM method [48]. For RR, RIA-VS achieves near-zero bias (−0.01 rpm) and 98.4% accuracy, while the head-motion [49] and chest-motion [50] methods exhibit larger biases (6.15 rpm and −5.27 rpm, respectively) and wider LoA. (**b**) Box plots of processing times indicate that RIA-VS has slightly higher mean execution times but provides more consistent and predictable performance with fewer extreme outliers.

To rigorously benchmark RIA-VS, we performed direct head-to-head comparisons with recently published state-of-the-art methods using our 15 s dataset. For HR estimation, RIA-VS was evaluated against a motion-robust POS-based method [47] and a learning-based CHROM filter-bank approach [48] on *n* = 199 samples. Bland–Altman analysis (Figure 13a) shows that RIA-VS substantially outperforms both methods, achieving 98.5% accuracy within ±10 bpm and markedly tighter LoA (+10.76 to −6.48 bpm), highlighting its superior robustness and precision. While the CHROM method exhibited improved stability compared with the POS-based pipeline yielding 84.9% accuracy and a low mean bias (−0.45 bpm), its LoA remained considerably wider (+28.88 to −29.79 bpm), indicating residual sensitivity to noise and inter-subject variability. In contrast, the POS-based method showed pronounced degradation at higher heart rates, with only 48.2% accuracy and a substantial positive bias (30.18 bpm), despite incorporating motion-state-aware adaptations. These findings suggest that extending chrominance projections alone, even with motion compensation or learning-based filtering, is insufficient to ensure reliable HR estimation from short facial recordings. Execution-time analysis (Figure 13b) further highlights this trade-off. Although the learning-based chrominance method achieved the fastest average runtime, RIA-VS delivered a more favorable balance between precision and computational predictability, avoiding the extreme variability and outliers observed in the POS-based pipeline. This consistency is particularly relevant for real-time or near-real-time clinical deployment.

For RR estimation, RIA-VS was benchmarked against head-motion [49] and chest-motion [50] based approaches using the same *n* = 191 samples that were used for RR analysis. Leveraging shoulder detection combined with vertical optical-flow analysis, RIA-VS achieved near-perfect accuracy (98.4% within ±5 rpm) and a negligible mean bias (−0.01 rpm). In contrast, both motion-based benchmarks demonstrated substantially lower reliability, with accuracies of 40.8% and 49.2%, respectively. The chest-motion method consistently underestimated RR (mean bias = −5.27 rpm), underscoring its susceptibility to posture, clothing, and non-respiratory motion artifacts. Collectively, these results demonstrate that RIA-VS provides superior robustness, accuracy, and consistency across both cardiovascular and respiratory domains, outperforming chrominance-based, learning-augmented, and motion-driven alternatives under identical short-duration recording conditions.

A key novel finding of this study is that the Q channel from the EVM-amplified YIQ video frames produces the highest SNR among all color channels evaluated, consistently approaching 80% and yielding a highly robust pulsatile signal. This enhancement arises because EVM selectively amplifies subtle cardiac-induced color variations, which are then captured by the Q channel while effectively suppressing noise from ambient lighting and motion. Unlike the luminance (Y) channel, which is highly sensitive to illumination changes, or the I channel, which encodes red–green contrast, the Q channel represents red-blue chrominance differences that closely track the cardiac cycle (Figure 5a). During systole, when arterial blood volume peaks, the skin appears slightly redder, whereas during diastole, when blood volume is minimal, it appears slightly bluer, producing a clear and physiologically meaningful pulsatile waveform (Figure 5b). By combining EVM with the Q-channel, our approach improves signal visibility and robustness, particularly in short 15 s recordings and across diverse skin tones (Figure 4), which is not achievable by methods that use the Q channel from raw RGB frames [51].

Other commonly used color spaces, including RGB, HSV, CMYK, LAB, XYZ, and YCbCr, fail to achieve comparable SNR (Figure 4) because they either mix physiological signals with overall luminance or encode color contrasts that are less sensitive to the subtle red-blue variations in blood volume. RGB channels are strongly affected by lighting and shadows. HSV and LAB separate hue and brightness in ways that do not isolate cardiac-related changes. CMYK and XYZ blend reflectance components, diluting the pulsatile signal, and YCbCr emphasizes luma-chroma separation that is misaligned with physiologically relevant color shifts. In contrast, the Q channel maximizes the contrast between systole and diastole while minimizing susceptibility to motion and illumination artifacts. This selective sensitivity enables minimal filtering, reduced computational load, and faster processing, all while maintaining very high HR estimation accuracy. By isolating true physiological signals from extrinsic noise, this approach overcomes the low-SNR limitations that have historically constrained non-contact rPPG, including individuals with darker skin tones, and provides a robust foundation for accurate, reliable vital-sign monitoring across diverse populations.

Complementing the high-fidelity HR estimation, this study introduces a novel shoulder-detection algorithm for RR estimation. By targeting anatomical regions that predominantly move with respiration, the method isolates breathing-induced motion while minimizing interference from non-respiratory movements such as limb shifts or torso adjustments. This precise ROI selection enables cycle-resolved RR estimation with exceptional accuracy. A key finding is the dual-reference validation framework, i.e., in addition to the traditional clinical reference $RR_{clinic}$ based on manual counting, we employed a cycle-level, video-observed reference $RR_{video}$ in which each breath was annotated in the video recordings. This temporally resolved reference provides a more accurate benchmark than conventional clinical counting, which is prone to inter-rater variability and temporal imprecision, often introducing discrepancies of ±6–8 rpm. The dual-reference validation not only confirms the intrinsic precision and robustness of the shoulder-motion method but also establishes a higher standard for assessing non-contact RR monitoring systems, ensuring reported performance reflects true physiological capture rather than artifacts from imprecise reference measurements.

The larger Bland–Altman LoA for RR when using the clinical reference versus the video-observed reference are likely to reflect, at least in part, the known inter- and intra-observer variability of manual RR counting in routine practice rather than deficiencies of the camera-based method itself. RR is a powerful but underutilized predictor of clinical deterioration, and several studies have shown that it is often either not measured or recorded inaccurately [52,53,54,55,56,57]. These findings highlight the importance of considering the quality of reference methods when evaluating new RR technologies, as a camera-based approach consistent with high-fidelity video reference may provide greater reliability than conventional manual counting in busy clinical settings. To quantify variability in the video-based reference $RR_{video}$, all recordings were independently annotated by a second physician, who counted respiratory cycles by observing the subject’s breathing movements in the video. Comparison with our original video-based counts allowed assessment of inter-rater reliability. Agreement between raters was excellent, with an intraclass correlation of 0.968 and a 95 percent confidence interval from 0.959 to 0.974, indicating near-perfect consistency. Bland–Altman analysis revealed zero mean bias and narrow LoA from −0.47 to +0.47 respiratory cycles, corresponding to less than half a breath difference between raters. These results confirm that video-based respiratory cycle annotation is highly reproducible, and that residual disagreement observed against the clinical reference $RR_{clinic}$ is largely due to inherent variability in live observation rather than limitations of the proposed method.

Although 15 s recordings delivered high-fidelity HR and RR estimates, the measured processing time (~68 s for HR) on standard home-grade hardware underscores a computational bottleneck. Importantly, prior work has demonstrated that rPPG pipelines can be dramatically accelerated through parallel computation and GPU use. For example, the pyVHR framework shows that an entire rPPG pipeline can run in real-time for 30 *f_s_* HD video on a GPU, with roughly a 5-fold speed-up compared to CPU processing [58]. Similarly, the RTrPPG network achieves inference times around 2.32 ms per frame on GPU (≈ 28.65 ms per frame on CPU), demonstrating that real-time HR estimation is feasible even for high-speed video streams [59].

These findings indicate that if our algorithm were ported to more capable hardware, e.g., a modern GPU-equipped workstation or dedicated server, processing times could be dramatically reduced, potentially to near real-time or low-latency operation. With parallel processing, optimized memory handling, and efficient implementation of filtering and signal extraction, the 15 s acquisition could be transformed into a continuous monitoring stream. Moreover, algorithmic optimizations such as sliding-window analysis, incremental signal processing, or adaptive frame-rate sampling could further decrease latency while preserving accuracy. In short, the computational overhead observed on a basic configuration hardware does not reflect a fundamental limitation of the method itself, but rather a practical challenge that can be overcome using well-established acceleration techniques.

Our findings extend previous proof-of-concept work from an emergency department cohort with suspected COVID-19, where a prototype camera-based system yielded mean vital-sign values close to reference methods but with substantial random variation [28]. If integrated into clinical workflows, a robust contactless HR and RR system could improve both the quality and frequency of vital-sign assessments in settings where time pressure and equipment constraints currently limit measurement. By reducing the need for repeated physical contact and shared devices, such systems may help decrease the risk of cross-infection between patients and staff, an issue highlighted during the COVID-19 pandemic but relevant for seasonal respiratory infections more broadly. Faster vital-sign acquisition could support triage of large numbers of patients with suspected infection, improving the likelihood of detecting the minority with serious illness. At the same time, the ability to obtain HR and RR without cuffs, probes, or auscultation may make monitoring more comfortable for patients and reduce the physical workload of nurses and physicians tasked with frequent observations. While our current study evaluated seated patients, the system is designed to accommodate supine, semi-recumbent, or otherwise non-seated individuals, and follow-up studies are planned to validate performance in varying clinical positions. In the longer term, camera-based monitoring might also enable self-assessment of vital signs in primary care clinics, pharmacies, or at home, provided that regulatory and usability requirements for unsupervised use can be met.

In addition to the anticipated clinical and workflow benefits, we have previously reported a cost-minimization analysis conducted in an emergency department setting that examined the measurement of five routinely assessed vital signs using camera-based methods [60]. That analysis suggested that non-contact measurement based on recorded facial videos could be more cost-effective than conventional approaches. Estimated annual cost savings exceeding €1 million were primarily attributed to reductions in staff time, consumables use, and device handling. The present study builds on this earlier work by providing further evidence of the technical accuracy and robustness of contactless HR and RR estimation, thereby strengthening the case for the potential clinical and system-level impact of scalable camera-based vital-sign monitoring in high-throughput care environments.

Despite the high agreement observed in this study, additional work is needed before widespread clinical implementation. We evaluated HR and RR in a relatively stable primary-care population under controlled lighting conditions; performance in unsupervised home settings, in patients with severe agitation or respiratory distress, and across a wider range of skin types still needs to be confirmed. Furthermore, we did not assess whether camera-based monitoring improves clinical decision-making, reduces time to recognition of deterioration, or affects hard outcomes such as hospitalization or mortality.

Building on the accuracy of HR and RR estimation demonstrated in the present study, and informed by our prior proof-of-concept work on contactless assessment of blood pressure and oxygen saturation [28], future work should explore the integration of additional physiological signals, such as blood oxygen saturation SpO_2_ and blood pressure, to expand the clinical relevance of non-contact monitoring. Incorporating SpO_2_ and blood pressure would enable comprehensive cardiorespiratory assessment from a single video source, supporting more sophisticated clinical decision-making and continuous patient monitoring in diverse healthcare settings. Ultimately, extending the system to measure multiple vital signs simultaneously would strengthen its applicability in complex workflows, including telemedicine, critical care, and home-based monitoring, and in general, supporting patients along their patient journey, creating a versatile tool for proactive and high-fidelity health assessment.

## 5. Conclusions

In conclusion, this study provides compelling evidence that camera-based, non-contact vital sign monitoring is both feasible and highly accurate. By leveraging the Q channel for robust rPPG extraction and a novel shoulder-motion tracking algorithm, the dual-modality system achieves exceptional performance, with HR and RR estimates reaching nearly 99% accuracy, respectively, from short 15 s recordings. The combination of rigorous video usability checks, cycle-level video references, and carefully optimized processing pipelines demonstrates that non-contact monitoring can overcome historical limitations such as low SNR, motion artifacts, and short-duration signal variability. Importantly, the results highlight the system’s adaptability to real-life scenarios, supporting rapid, reliable vital sign assessment without physical contact. Collectively, these findings establish a strong foundation for clinical translation, paving the way for scalable, non-invasive monitoring solutions in hospitals, outpatient care, and home-based health management, and anywhere along the patient journey with the use of telemedicine applications.

## Figures and Tables

**Table 1:** Accuracy of the proposed non-contact HR and RR algorithm across multiple tolerance thresholds. Values indicate the percentage of estimates within ±1 and ±10 bpm for HR, and ±1 and ±5 rpm for RR, highlighting the precision of the method at different allowable deviations.

			Accuracy % vs. Tolerance Thresholds				
	Duration	Avg. Processing Speed	±1	±3	±5	±8	±10
HR	15 s	68.3 s	26.6	66.8	91.4	96.5	98.5
	10 s	38.8 s	25.7	61.3	86.1	93.9	95.6
	5 s	16.9 s	21.2	48.1	69.3	84.1	89.6
	Duration	Avg. Processing Speed	±1	±2	±3	±4	±5
$RR_{clinic}$	15 s	41.8 s	29.8	54.4	71.7	79.6	89.0
	10 s	25.0 s	23.8	44.5	61.7	74.7	83.1
$RR_{video}$	15 s	41.8 s	93.7	97.4	97.4	98.4	98.4
	10 s	25.0 s	55.7	79.6	88.4	92.3	95.1

## Data Availability

The clinical videos used in this study were collected under ethical approval from the Swedish Ethical Review Authority, with written informed consent obtained from all participants. Due to privacy concerns and institutional restrictions, the raw facial video data are not publicly available. However, signal-level datasets generated from the video analysis may be made available by the corresponding author upon reasonable request and subject to a data-sharing agreement and approval from the relevant ethics committee.

## Abbreviations

The following abbreviations are used in this manuscript:

HR	Heart rate
RR	Respiratory rate
s	seconds
rPPG	remote photoplethysmography
SNR	signal-to-noise ratio
ROI	Region of interest
EVM	Eulerian video magnification
RGB	Red-Green-Blue
MAE	mean absolute error
bpm	beats per minute
rpm	respirations per minute
LoA	limits of agreement
*f_s_*	frames per second
HSV	Hue Saturation Value
CMYK	Cyan Magenta Yellow Key
RMSE	root-mean-square error
POS	plane-orthogonal-to-skin

## Appendix A

### Pseudocode for Non-Contact HR and RR Estimation

Input: High-speed RGB video of the subject Output: HR, RR, error flags Step 1: Video quality assessment 1. Load video. 2. Sample frames at regular intervals. 3. For each frame: Convert to grayscale. Compute mean intensity → detect low illumination. Compute absolute difference from previous frame → detect flicker. Apply Laplacian filter → compute variance → detect blur. 4. If flicker, low illumination, or blur → mark video as invalid and stop processing. Step 2: Face detection 1. Apply face detector to the first frame. 2. If no face detected → stop processing. Step 3: Shoulder detection for RR 1. Extract the lower quarter of the frame. 2. For left and right sides: Convert to grayscale. Detect edges using Sobel operator. Flip vertically → detect topmost edges of shoulder region. Fit line to top edge points → compute slope. Shoulder is visible if slope < 45°. 3. If neither shoulder is visible → unable to compute RR, return error. Step 4: Face tracking and respiratory motion estimation 1. Detect feature points (corners) in the face region. 2. Initialize KLT point tracker with these points. 3. For each frame: Track points using Lucas–Kanade optical flow. Estimate geometric transformation (similarity) → update bounding box. If face moves beyond threshold → motion detected, return error. Else, crop face region and store for rPPG extraction. Apply Gaussian blur to reduce skin texture artifacts. Every 1/3 s: Crop shoulder region. Convert to grayscale. Compute vertical motion using optical flow. Average vertical motion → append to respiratory signal. 4. Smooth respiratory signal and remove outliers. 5. Identify peaks and valleys → count respiratory cycles. 6. Compute RR in cycles per minute. Step 5: Eulerian alpha ($\alpha_{l}$) calculation 1. Convert first face frame to HSV. 2. Extract intensity (V channel). 3. Compute histogram → locate maximum intensity. 4. Set $\alpha_{l}$ Bright skin relative to illumination (intensity > 0.5) → higher $\alpha_{l}$ Dark skin relative to illumination (intensity ≤ 0.5) → lower $\alpha_{l}$ 5. This adapts EVM amplification for skin tone. Step 6: Eulerian video magnification for rPPG 1. Build Gaussian pyramid of face images. 2. Apply temporal bandpass filter. 3. Amplify filtered images by$\;\alpha_{l}$ 4. Reconstruct amplified frames. 5. Convert frames to YIQ colour space → extract Q channel. 6. Average pixel intensity of Q channel → raw rPPG signal. Step 7: HR extraction 1. Remove outliers from rPPG signal. 2. Smooth signal using moving average and Gaussian filter. 3. Detect systolic peaks. 4. Compute HR in beats per minute. 5. Store HR value. Step 8: Save outputs 1. Save HR, RR, and error flags. 2. Log error types if processing failed at any stage. Step 9: Error handling 1. Face not found 2. Shoulders not found 3. Face has moved 4. Flickering detected 5. Low illumination 6. Blurred recording

Key parameters used in the HR and RR estimation workflow are summarized in Table A1 and were empirically optimized for performance.

**Table A1:** List of parameters for HR and RR estimation.

Parameter	Value	Unit	Description
Video frame rate	390	*f_s_*	Acquisition rate of high-speed camera
Flicker threshold	2	gray intensity units	Max allowed frame-to-frame intensity change
Low-illumination threshold	80	gray intensity units	Frames below this intensity are considered dark
Blur variance threshold	4	a.u.	Laplacian variance threshold for blur detection
Shoulder slope threshold	45	degrees	Max slope to consider shoulder visible
Optical flow threshold	0.0005	a.u.	Lucas-Kanade noise threshold
Face movement threshold	10	pixels	Max allowed displacement between frames
Gaussian blur σ	8	pixels	Smoothing for face images
Eulerian pyramid levels	6	a.u.	Number of Gaussian pyramid levels for EVM
$\mathrm{EVM}\;\alpha_{l}$ (for bright skin)	150	a.u.	Amplification factor for bright skin
$\mathrm{EVM}\;\alpha_{l}$ (for dark skin)	90	a.u.	Amplification factor for dark skin
Temporal bandpass low	50	bpm	Lower cutoff for HR bandpass filter
Temporal bandpass high	200	bpm	Upper cutoff for HR bandpass filter
Outlier removal	2-98	percentiles	Percentiles used to remove extreme values
Smoothing windows	*f_s_*/4, *f_s_*/2	frames	Moving average and Gaussian smoothing for rPPG
RR sampling interval	1/3	s	Interval at which optical flow is computed
